# Integrated Deadenylase Genetic Association Network and Transcriptome Analysis in Thoracic Carcinomas

**DOI:** 10.3390/molecules27103102

**Published:** 2022-05-12

**Authors:** Athanasios Kyritsis, Eirini Papanastasi, Ioanna Kokkori, Panagiotis Maragozidis, Demetra S. M. Chatzileontiadou, Paschalina Pallaki, Maria Labrou, Sotirios G. Zarogiannis, George P. Chrousos, Dimitrios Vlachakis, Konstantinos I. Gourgoulianis, Nikolaos A. A. Balatsos

**Affiliations:** 1 Department of Biochemistry and Biotechnology, University of Thessaly, Biopolis, 415 00 Larissa, Greece; thanoskyrit@hotmail.com (A.K.); eirini.papanastasi@unil.ch (E.P.); pamarago@hotmail.com (P.M.); d.chatzileontiadou@latrobe.edu.au (D.S.M.C.); lipal@hotmail.gr (P.P.); maria_labrou@hotmail.com (M.L.); 2 Department of Respiratory Medicine, Faculty of Medicine, University of Thessaly, Biopolis, 411 10 Larissa, Greece; kouirouxis@hotmail.gr; 3 Department of Pneumonology-Oncology, Theagenio Cancer Hospital, 540 07 Thessaloniki, Greece; 4 Department of Physiology, Faculty of Medicine, University of Thessaly, Biopolis, 415 00 Larissa, Greece; 5 University Research Institute of Maternal and Child Health and Precision Medicine, ‘Aghia Sophia’ Children’s Hospital, National and Kapodistrian University of Athens, 115 27 Athens, Greece; chrousos@gmail.com (G.P.C.); dimvl@aua.gr (D.V.); 6 UNESCO Chair on Adolescent Health Care, ‘Aghia Sophia’ Children’s Hospital, National and Kapodistrian University of Athens, 115 27 Athens, Greece; 7 Center of Clinical, Experimental Surgery and Translational Research, Division of Endocrinology and Metabolism, Biomedical Research Foundation of the Academy of Athens, 115 27 Athens, Greece; 8 Laboratory of Genetics, Department of Biotechnology, School of Applied Biology and Biotechnology, Agricultural University of Athens, 118 55 Athens, Greece

**Keywords:** deadenylases, lung cancer, association network, transcriptomics, mRNA degradation

## Abstract

The poly(A) tail at the 3′ end of mRNAs determines their stability, translational efficiency, and fate. The shortening of the poly(A) tail, and its efficient removal, triggers the degradation of mRNAs, thus, regulating gene expression. The process is catalyzed by a family of enzymes, known as deadenylases. As the dysregulation of gene expression is a hallmark of cancer, understanding the role of deadenylases has gained additional interest. Herein, the genetic association network shows that CNOT6 and CNOT7 are the most prevalent and most interconnected nodes in the equilibrated diagram. Subsequent silencing and transcriptomic analysis identifies transcripts possibly regulated by specific deadenylases. Furthermore, several gene ontologies are enriched by common deregulated genes. Given the potential concerted action and overlapping functions of deadenylases, we examined the effect of silencing a deadenylase on the remaining ones. Our results suggest that specific deadenylases target unique subsets of mRNAs, whilst at the same time, multiple deadenylases may affect the same mRNAs with overlapping functions.

## 1. Introduction

The poly(A) tail is a key element that determines mRNA stability and is central to the regulation of gene expression. The shortening and removal of the tail by specific 3′ exoribonucleases, known as deadenylases, is the first and rate-limiting step of mRNA degradation [1,2]. At least 10 deadenylases were characterized in humans, classified into two families; DEDD (after the conserved Asp and Glu residues in the active site) and the EEP (exonuclease–endonuclease–phosphatase) [1]. Deadenylases may act in multi-subunit complexes, such as the carbon catabolite repressor protein 4 (CCR4)-negative on TATA (NOT) complex (CCR4–NOT or CNOT) [3], in heterocomplexes, including the Pan2–Pan3 complex consisting of a 1:2 stoichiometry [4,5], and oligomeric, such as the poly(A)-specific ribonuclease (PARN) [6,7]. The CCR4–NOT complex is the dominant deadenylase complex in humans and flies. It consists of two deadenylases (CCR4 and CAF1) and non-catalytic subunits, including the conserved NOT1, NOT2, NOT3, and CAF40 [3]. The complex is characterized by the presence of one deadenylase from each family; CNOT7 or CNOT8 (DEDD), and CNOT6 or CNOT6L (EEP), respectively. The presence of multiple deadenylases raises several questions: whether they act on specific mRNA subsets, or multiple deadenylases target the same mRNA, with discrete but overlapping functions [1]. These suggest that the control of mRNA turnover is dictated by the regulation of the activity of these enzymes, as well as by factors that recruit deadenylases in their targets, including proteins and microRNAs [2]. Further, it was proposed that deadenylases act in concert; PAN2 deadenylase initiates the shortening of a reporter β-globin mRNA in mouse NIH3T3 fibroblasts poly(A) tail to roughly half the length, while CNOT6 (Ccr4a) completes the removal, triggering the degradation of the entire mRNA [8]. Another question is the timing of the deadenylation and subsequent degradation; several microRNAs that recruit deadenylases to target RNAs for degradation are rhythmic, yet the knowledge on the time-dependent activation of deadenylases, and mRNA degradation under time-keeping phenomena, is very limited and only one circadian deadenylase has been characterized so far [9].

Several studies highlight the role of deadenylases in cancer, when gene expression is dysregulated [10,11,12]. Thus, CNOT6L modulates the levels of p27Kip1, a tumor suppressor that also functions as an oncogene [12,13,14]. CNOT7 and the subsequent transcriptional decay were proposed to determine progression, further suggesting that targeting the deadenylase activity might propose novel therapy [15]. PARN is activated by the tumor suppressor BARD1 and may, in turn, act as tumor suppressor, destabilizing c-fos, c-jun, IL-8, uPA, VEGF, and TNF-alpha mRNAs [16,17,18,19,20], while in concert with the miR-125b that regulates the levels of p53 mRNA [21]. In line with the latter, the depletion of PARN in cells of gastric cancer origin is associated with increased levels of p53 [22].

Regarding the levels of deadenylases in cancer, it is shown that in acute types of leukemia (AML, ALL), the levels of both PARN and CNOT6 increase compared to non-malignant clinical samples, while the opposite is the case for CNOT6L and CNOT7 [23]. SNPs in the CNOT6 gene are significantly associated with B-cell ALL susceptibility [24], and a significant loss in the CNOT6L copy number is reported in human colon adenocarcinoma samples [25]. The expression of CNOT8 is elevated in primary colorectal carcinoma and metastatic legions compared to the normal mucosa [26]. Regarding lung cancer, it is reported that in clinical samples from SCC, the expression of PARN, CNOT6, and Nocturnin is associated with overall survival, while CNOT6 overexpression is a strong indicator against metastasis, thus highlighting deadenylases as promising survival and prognostic factors in lung cancer [27]. Of note, Nocturnin (NOC) is now considered as a phosphatase, instead of a deadenylase [28]. The previous observations imply that deadenylases both regulate the stability of important cancer-related mRNAs, and that their levels vary in cancer subtypes, suggesting that they might be used as biomarkers, with diagnostic and prognostic values.

In this work, based on our previous study of deadenylases in SCC, we investigated the impact of PARN and the members of the CNOT complex, CNOT6, CNOT6L, CNOT7, and CNOT8 on gene expression. We designed a genetic association network, silenced each one of these enzymes in two cell lines (NCI-H520 of SCC origin and HEp-2 cells used in studies on lung cancer, and analyzed the changes in gene expression with cDNA microarrays. We extended the analysis into a third cell line, MCF7, previously used to study the impact of CNOT deadenylases on global gene expression. Several transcripts and gene ontologies are regulated by specific deadenylases, while CNOT8 is not expressed in NCI-H520 cells. We also examined the impact of silencing a deadenylase on the expression levels of the other enzymes. Our results suggest that specific deadenylases target unique subsets of mRNAs, while at the same time multiple deadenylases act on the same mRNA with overlapping functions. A deadenylase affects the levels of the other enzymes, and may also compensate for members that are not expressed.

## 2. Results

### 2.1. Genetic Association Studies for CNOT6, CNOT6L CNOT7, CNOT8, and PARN

The previous observations on the emerging role of deadenylases in gene expression in cancer highlight the need for the investigation of genetic interplay of key genes in the disease. Similar gene expression patterns [29], the interaction between gene products of known genes [30], and similar phylogenetic profiles [31] are indications of similar functions of these genes. The existing algorithms used for predicting gene function mainly use the guilt-by-association principle. Their application involves adding related genes to a “seed-list” of functionally known genes. These algorithms typically compute a “functional correlation network” to represent each dataset, where each node corresponds to proteins or genes, and the ends are weighted according to co-functionality data. The kernels used by support vector machines (SVMs) [32,33], functional association networks [34], and protein-protein association maps [35] are three types of functional association networks. Individual association networks are often combined to create a complex association network that summarizes all indications of co-functionality. The predictions of these algorithms are quite accurate in the case of non-annotated genes, since using multiple complementary data sources means they are able to accurately predict previously annotated gene functions blind [36]. In the case of the genes in this study, namely, CNOT6, CNOT6L CNOT7, CNOT8, and PARN, the genetic network returns quite intriguing associations.

The guilt-by-association genetic networks, despite their advantages, are not yet widely used for gene annotation or new hypotheses about gene function. Centrally managed web-based “prediction servers” are an effective strategy for ensuring access to available predictions, since collecting large numbers of heterogeneous data sources, creating functional association networks to represent these sources, and mapping gene identifiers between networks is a complex process. However, most predictive servers promote speed while sacrificing accuracy, and their predictions are stored in static databases that can easily become outdated [37,38].

Label propagation algorithms, such as the Gaussian field label propagation algorithm [39], are used to predict gene function from the complex network. Like most prediction algorithms, these algorithms rate each node on the network at a so-called “discrete value”, which is a threshold for making predictions, as it reflects the calculated degree of correlation that the node has in the seed list, which determines the given function. In addition to the Gaussian field tag diffusion algorithm, there is a wide variety of label propagation algorithms, such as those based on functional flow [40] or Markov random fields [41].

**Figure 1:**
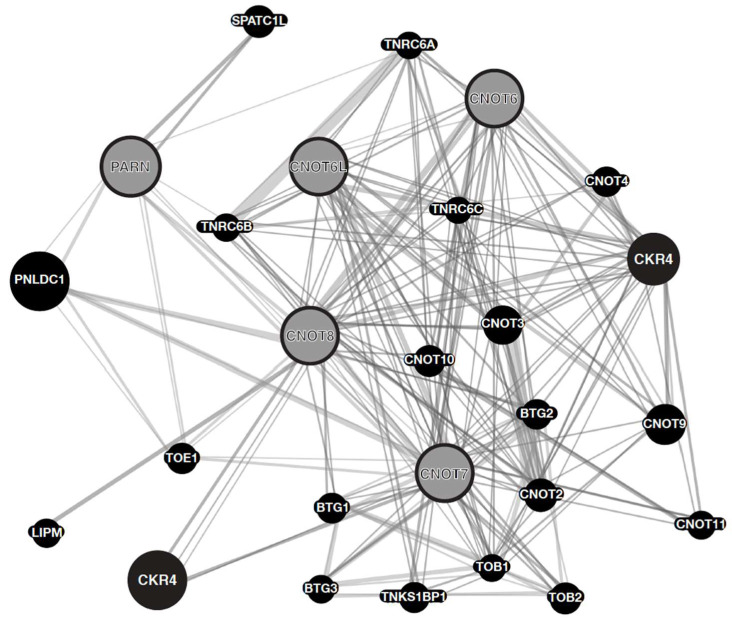
Genetic association network for CNOT6, CNOT6L CNOT7, CNOT8, PARN deadenylases. The genetic network shows the most frequent neighboring genes. Every gene is represented as a node (query genes are in grey color). Genes are linked by associated networks that include co-expression, physical contact, co-localization, genetic interactions, pathways, and shared protein domains. Lines represent the abovementioned different interactions between the gene nodes.

Based on the previous study, the genetic association network for the CNOT6, CNOT6L CNOT7, CNOT8, and PARN genes was designed. The aim was to exploit both the large amount of data, and the variety of genetic and proteomic data, to predict the functions of known genes by in silico techniques. The genetic network was constructed with GeneMANIA, and demonstrates that the CNOT deadenylases form an association network, while PARN seems to associate with CNOT8 (Figure 1). The designed genetic network provides invaluable information on the crosstalk and interplay between those genes. Depending on the mode of association of the abovementioned genes (co-expression, pathway, physical contact, or co-localization), the number of lines/interactions between sets of genes pinpoints the significance of each node (gene) on the constructed network. However, since this is a computational approach, the unique interplay and crosstalk amongst the CNOT6, CNOT6L, CNOT7, CNOT8, and PARN genes is confirmed and further investigated. Based on the findings of the genetic network, it is noticeable that CNOT6 and CNOT7 are the most prevalent and most interconnected nodes in the equilibrated diagram. In this direction, a microarray analysis was sought to corroborate the expression levels and interactions observed in our in silico study.

### 2.2. Each Deadenylase Has a Limited Number of Specific Target Transcripts

According to the genetic network, we examined the association between CNOT deadenylases and the impact on gene expression in two cell lines of different cancer origin, NCI-H520, of squamous cell lung carcinoma, and HEp-2, originally derived from human epidermoid (Hep) carcinoma of the larynx [42,43]. NCI-H520 (H520) cells were isolated from the lung tissue of a male squamous cell carcinoma patient. Genetic alterations, including the mutation of the epidermal growth factor receptor, or v-Ki-ras2 kirsten rat sarcoma viral oncogene homolog, and the fusion of anaplastic lymphoma kinase (*ALK*), RET proto-oncogene (*RET*), or v-ros UR2 sarcoma virus oncogene homolog 1 (*ROS1*), occur in non-small cell lung cancers [44]. A search conducted in the Expasy database for mutations in NCI-H520 rendered three mutated genes: ATM, CDKN2A, and TP53 (https://web.expasy.org/cellosaurus/CVCL_1566) (accessed on 5 April 2022). We extended our search for mutations in COSMIC (Catalogue Of Somatic Mutations In Cancer; https://cancer.sanger.ac.uk/cosmic (accessed on 10 February 2022) [45]. The search of the COSMIC Cell Line Gene Mutation Profiles dataset rendered at least 445 mutated genes from NCI-H520 cells; https://cancer.sanger.ac.uk/cell_lines/sample/overview?id=908443 (accessed on 10 February 2022) [46]. The sequencing of the p53 cDNA for HEp-2 shows no mutations, while the cells produced HPV 18/E6-inactivated protein [47].

**Figure 2:**
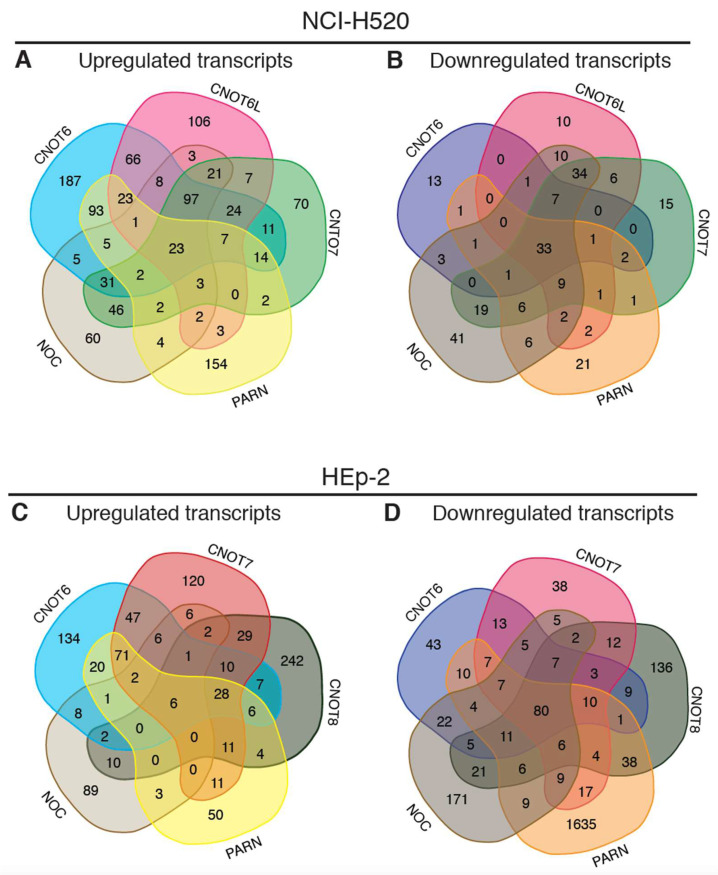
Transcripts affected by each deadenylase in NCI-H520 and HEp-2 cells. (**A**) Venn diagrams showing upregulated transcripts of CNOT6, CNOT6L, CNOT7, PARN, and NOC KD in NCI-H520 cells. (**B**) Venn diagrams showing downregulated transcripts of CNOT6, CNOT6L, CNOT7, PARN, and NOC KD in in NCI-H520 cells. (**C**) Venn diagrams showing upregulated transcripts of CNOT6, CNOT7, CNOT8, PARN, and NOC KD in HEp-2 cells. (**D**) Venn diagrams showing downregulated transcripts of CNOT6, CNOT7, CNOT8, PARN, and NOC KD in HEp-2 cells. The numbers represent differentially expressed transcripts, the levels of which are altered by FC ≥ 2.0 upon silencing of the indicated deadenylase.

The combination of the observations on gene expression alterations in SCC upon silencing of the deadenylases of the CCR4-NOT complex with previous results of PARN silencing [27] reveal transcripts that are differentially expressed upon deadenylase silencing. Subsequently, we performed genome-wide expression analysis to identify possible target transcripts upon a shRNA mediated knockdown of CNOT6, CNOT6L, CNOT7, PARN, and NOC. NOC was originally described as a deadenylase, yet it is currently considered as a phosphatase [28]. Nevertheless, we included NOC in our analysis, as it was studied in the regulation of several biological processes and gene expression studies, including lung cancer [27,48,49]. CNOT8 expression is not detectable in NCI-H520 cells by RT-qPCR, and was not included in our analysis. Upon the silencing of CNOT6, CNOT6L, CNOT7, PARN, and NOC in NCI-H520 cells, we detect 582, 379, 360, 323, and 313 transcripts being upregulated more than two-fold (fold-change 2; FC ≥ 2.0), respectively (Figure 2A, Table S1). In addition, only some mRNAs show decreased levels in CNOT6, whereas 114, 135, 85, and 171 mRNAs decrease in CNOT6L, CNOT7, PARN, and NOC in NCI-H520 knockdown (KD) cells, respectively, compared to their controls (Figure 2B, Table S1). We also detect 23 upregulated genes and 33 downregulated genes, common across all 5 deadenylases knockdown experiments (Figure 2A,B). The transcripts that are deregulated upon the silencing of only one deadenylase are possible exclusive targets of that deadenylase.

To obtain more insight into the molecular functions and biological processes of the identified differentially expressed transcripts, we performed over-representation analysis (ORA) of gene ontology (GO) categories using the ConsensusPathDB-human online tool (http://consensuspathdb.org/ (accessed on 21 March 2021) [50,51]. The analysis of the common upregulated transcripts reveals 30 enriched GOs, while of the common downregulated transcripts reveals 21 enriched GOs (Table S2).

We have previously shown that PARN and NOC affect diverse mRNA populations in different cell lines [27]. Thus, HEp-2 cells were treated with shRNAs targeting CNOT6, CNOT7, CNOT8, PARN, and NOC. CNOT6L is not efficiently silenced in HEp-2 cells, and, thus, its impact is not examined. Microarray analysis results in 1406 upregulated transcripts, with only 6 transcripts common among deadenylases. Further, among 3037 downregulated transcripts, 80 are common (Figure 2C,D, Table S3). Following deadenylases silencing, only 3 GOs are predicted to be enriched by the common upregulated transcripts, and 18 GOs by the common downregulated transcripts (Table S4).

Taken together, the silencing of deadenylases in two cell lines of different cancer origin (NCI-H520 and HEp-2) affects different groups of transcripts with very few genes in common, suggesting a discrete role for each enzyme in different cancer types. It seems that the levels and the stability of the majority of transcripts are meticulously controlled, as very few transcripts are regulated by a single deadenylase.

### 2.3. Deadenylase Silencing–Comparison with MCF7 Cell Line

**Figure 3:**
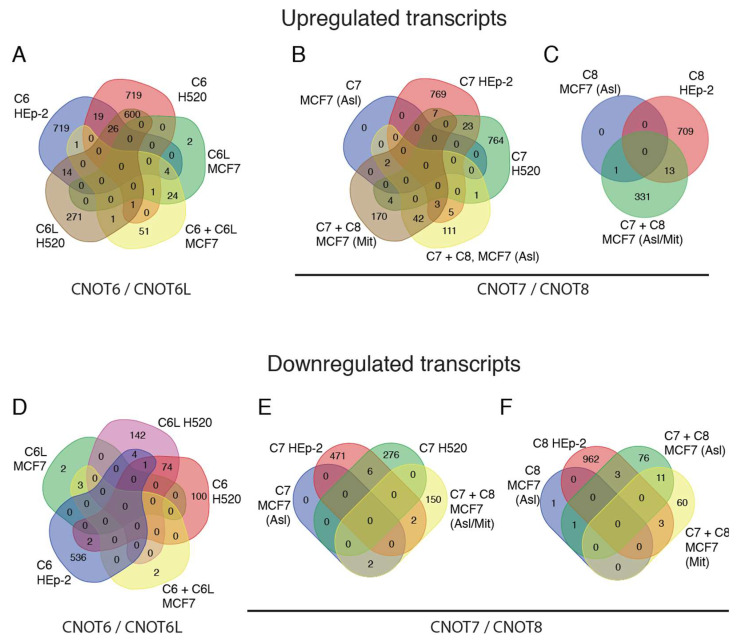
Upregulated and downregulated transcripts upon deadenylase silencing in NCI-H520, HEp-2, and MCF7 cells. (**A**–**C**) Venn diagrams showing upregulated transcripts after silencing of CNOT6 and CNOT6L, CNOT7 and CNOT8 in NCI-H520, HEp-2, and MCF7 cells. (**A**) Silencing of CNOT6 and CNOT6L in NCI-H520, HEp-2, and MCF7 cells, and in combination with CNOT6L (C6 + C6L) in MCF7 cells. (**B**) Silencing of CNOT7 in NCI-H520, HEp-2, and MCF7 cells and in combination with CNOT8 (C7 + C8) in MCF7 cells. (**C**) Silencing of CNOT8 in HEp-2 and MCF7 cells, and in combination with CNOT7 (C7 + C8) in MCF7 cells. (**D**–**F**). Venn diagrams showing downregulated transcripts after silencing of CNOT6, CNOT6L, CNOT7, and CNOT8 in NCI-H520, HEp-2, and MCF7 cells. (**D**) Silencing of CNOT6 and CNOT6L in NCI-H520, HEp-2, and MCF7 cells, and in combination (C6 + C6L) in MCF7 cells. (**E**) Silencing of CNOT7 in NCI-H520, HEp-2, and MCF7 cells, and in combination with CNOT8 (C7 + C8) in MCF7 cells. (**F**) Silencing of CNOT8 in HEp-2 and MCF7 cells, and in combination with CNOT7 (C7 + C8) in MCF7 cells. The numbers represent differentially expressed transcripts, the levels of which are altered by FC ≥ 1.5 upon silencing of the indicated deadenylase. The data for transcripts from MCF7 cells are from the studies of Aslam et al. and Mittal et al. [52,53]. Asl, Aslam; Mit, Mittal; Asl/Mit, Aslam/Mittal, respectively. C6, CNOT6; C6L, CNOT6L; C7, CNOT7; C8, CNOT8.

To extend the previous analysis, we included data from silencing of CNOT6, CNOT6L, CNOT7, and CNOT8 from another cell line, namely, MCF7 (derived from pleural effusion of breast carcinoma), based on previous studies [52,53]. In the studies of Aslam and coworkers, and Mittal and coworkers, the analysis is based on 1.5-fold changes (FC ≥ 1.5) of the levels of transcripts. To acquire comparable results, we modified our analysis on NCI-H520 and HEp-2 cells, calculating alterations in the transcript levels by FC ≥ 1.5. Upon the silencing of CNOT6 in NCI-H520 and HEp-2, and compared to the silencing of CNOT6/CNOT6L in MCF-7 cells, we found 45 common upregulated genes between NCI-H520 and HEp-2, 2 between CNOT6 KD NCI-H520 and CNOT6/CNOT6L KD MCF7 cells (GPR81, DCLK1), and 5 between HEp-2 and CNOT6/CNOT6L KD MCF7 cells (LMO3, MUCL1, SLITRK6, GALNT12, and F7). We find only three common down regulated genes in NCI-H520 and HEp-2 cells (LYPD1, GPR153, and RBM24). Our analysis also reveals only two common upregulated genes in CNOT6L KD NCI-H520 and CNOT6/CNOT6L KD MCF7 cells (CXCL17, GPR81), but no common downregulated transcripts among CNOT6L KD NCI-H520, HEp-2, and MCF7 cells. CNOT7 KD in HEp-2 results in 23 common upregulated with NCI-H520, and 15 in CNOT7/CNOT8 KD MCF-7 cells, while CNOT7 KD in NCI-H520 reveals only 5 common upregulated genes compared to CNOT7/CNOT8 KD MCF-7 cells (GRHL3, BLNK, MAP2, IL4R, and COL27A1). Less common downregulated transcripts are found upon CNOT7 silencing between HEp-2 and NCI-H520, and HEp-2 and CNOT7/CNOT8 KD MCF-7 cells. Only 13 common upregulated, and 6 downregulated, genes are found between HEp-2 and CNOT7/CNOT8 KD MCF7 cells (Table S5). We do not identify any common deregulated transcripts among the three cells lines. The results of the upregulated and downregulated transcripts of the above-mentioned silencing experiments are summarized in the Venn diagrams presented in Figure 3. Taken together, these results suggest that a very limited number of transcripts are commonly affected among three cell lines, while the absence of any common target suggests that deadenylases target specific transcripts, likely through differential regulation and recruitment in a tissue-dependent manner.

### 2.4. Non-Coding RNA Genes Affected by Deadenylases

**Figure 4:**
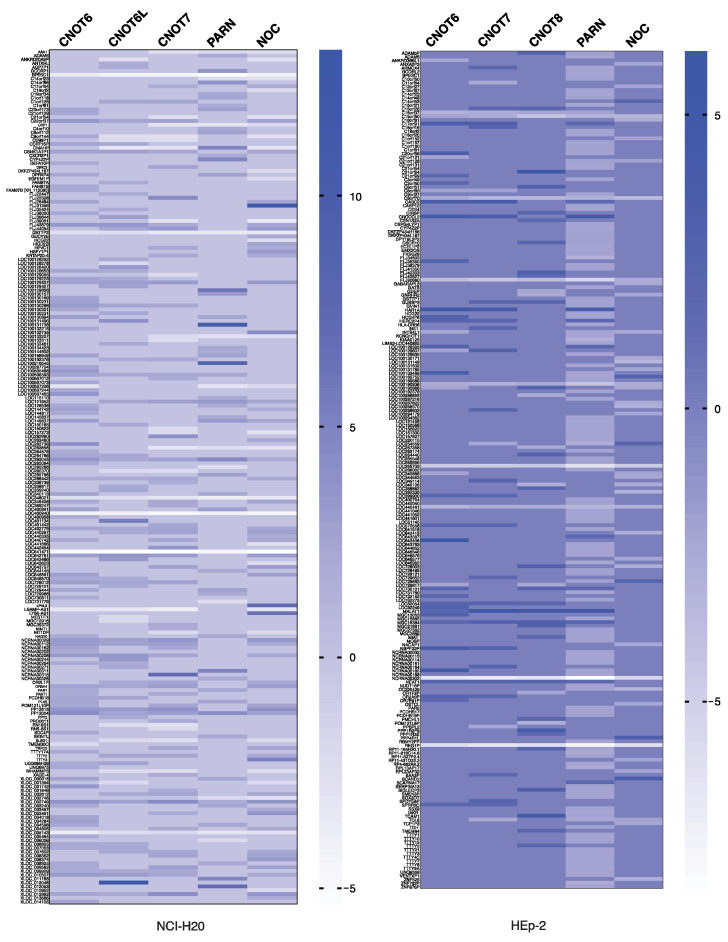
Non-coding RNA transcripts ffected by deadenylase silencing in NCI-H520 and HEp-2 cells. Heat map of differentially expressed non-coding RNA genes (FC ≥ 2.0) upon shRNA-mediated silencing of CNOT6, CNOT6L, CNOT7, PARN, and NOC expression.

Several of the genes affected by the silencing of deadenylases presented above encode for non-coding RNAs. In NCI-H520 cells, BPESC1 lncRNA, GSTTP2 non-coding RNA, LOC348021 (LINC00442) long intergenic non-coding RNA, LOC400940 lncRNA, LOC541471 lncRNA, and XLOC 005143 are all downregulated upon the silencing of deadenylases and NOC. Several non-coding RNAs are upregulated upon PARN silencing, including LOC100129858, LOC100131738, LOC100216545, XLOC011185, and NCRNA00311. Furthermore, upregulated non-coding RNAs include LOC401134 and XLOC012046 when the CNOT6L expression is impaired, and NCRNA00315 upon CNOT7 silencing. The results are summarized in Figure 4 and Table S6. In HEp-2 cells, LOC100190938, C9orf70, REG1P, C15orf50, NCRNA00202, LOC440461, LOC285733 non-coding RNAs, and FLJ90680 miscRNA are all downregulated upon silencing of the deadenylases and NOC. Only three non-coding RNAs are upregulated upon the silencing of deadenylases; MGC16384, NEAT1, and MALAT1. The Table S7 summarizes the results of silencing in HEp-2 cells, which are also depicted in the heat map in Figure 4. Overall, the silencing of the examined deadenylases impairs the expression of non-coding RNA genes, while a limited number of these seem to represent common targets of the enzymes.

### 2.5. The Silencing of One Deadenylase Affects the Levels of Other Deadenylases

**Figure 5:**
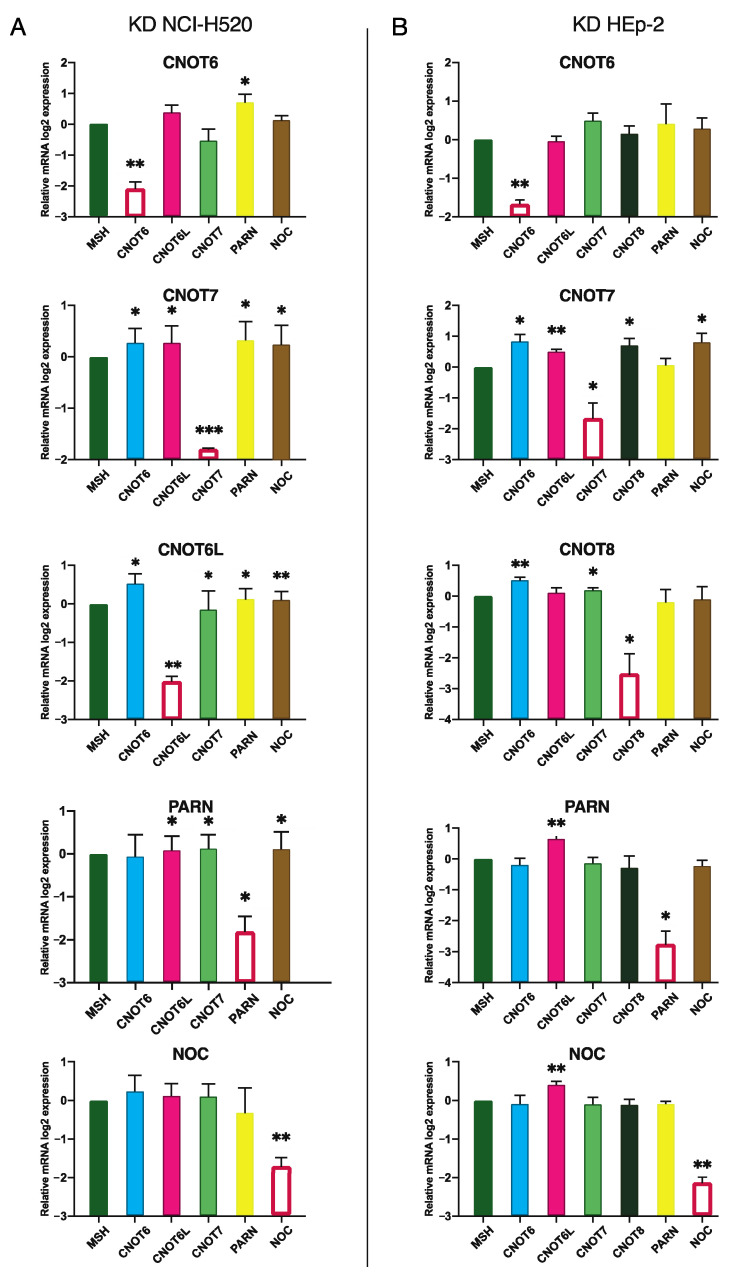
The silencing of a deadenylase affects the expression of other deadenylases. The levels of deadenylases were measured with RT-qPCR analysis following silencing of one deadenylase at a time in NCI-H520 (**A**) and in HEp-2 cells (**B**). The levels of the silenced enzyme are indicated with a red-outlined white column. MSH, control levels from cells transfected with non-targeting shRNA. * *p* ≤ 0.05; ** *p* ≤ 0.01.

Finally, as it was proposed that deadenylases may have overlapping functions and their action is concerted [1,8,54], we examined whether the silencing of a single deadenylase affects the levels of other deadenylases. Hence, PARN, CNOT6, CNOT7, or CNOT6L, were knocked down one at a time in NCI-H520 cells, and the levels of the remaining deadenylase mRNAs were determined with RT-qPCR. CNOT6 silencing results in upregulation of PARN. CNOT6 itself shows increased levels upon CNOT6L or CNOT7 KD. PARN seems to upregulate the expression of the other deadenylases, except the expression of CNOT6. For all the other conditions examined, the analysis demonstrates that the expression of the remaining deadenylases is unaffected (Figure 5A). We were not able to detect any CNOT8 expression in NCI-H520 cells.

In HEp-2 cells, CNOT6L levels increase upon CNOT7, PARN, or NOC silencing. We observe CNOT6 and CNOT7 upregulation following CNOT8 silencing, while CNOT7 KD results in increased NOC levels. In contrast to NCI-H520 cells, PARN is not affected by the silencing of the other deadenylases (Figure 5B).

Previous studies report that the knockdown of CNOT6 is (partially) compensated by CNOT6L, as well as CNOT7 by CNOT8 in MCF7, cells due to their high sequence similarity [52,53]. Herein, we observe that CNOT7 affects CNOT8 expression and vice versa, possibly acting in a negative feedback regulatory loop. Unexpectedly, the silencing of CNOT6L increases CNOT6 levels, whilst CNOT6L expression is unaffected upon CNOT6 KD. 

Conclusively, it seems that certain deadenylases modulate the expression of the majority of the other deadenylase, such is the case for CNOT7, while others, such as CNOT6, have minor impact. Further, each deadenylase is differently affected by the other deadenylase between the two cell types, implying tissue-specificity regulation.

## 3. Discussion

Worldwide, lung cancer is the most common cause of major cancer incidence and mortality, primarily because it is detected in an advanced stage for both sexes. The search for biomarkers for diagnosis, prognosis, and therapeutic purposes is of primary importance. In this direction, any strategy to improve prediction, treatment outcome, and early detection and classification has to focus on the mechanisms of development and progression of the disease. In this work, we studied the impact of deadenylases on gene expression in cells of lung cancer origin.

In this work, we investigated the impact of several deadenylases on gene expression in cell lines of cancer origin. We designed a genetic association network for PARN, and the deadenylases of the CNOT complex, CNOT6, CNOT6L, CNOT7, and CNOT8. CNOT6 and CNOT7 are the most prevalent and most interconnected nodes in the equilibrated diagram (Figure 1). Our transcriptomic analysis identifies transcripts possibly regulated by specific deadenylases, whilst a limited number of genes are commonly regulated by all the deadenylases examined. The common deregulated transcripts are involved in several pathways and biological processes, including synaptic signaling, transmembrane transport, detection of chemical stimulus, and cell death. We extended the analysis to a third cell line, MCF7, from a different cancer type, that was previously used to study the impact of deadenylase impact on global gene expression [52,53]. An important observation of this analysis is that no transcript seems to be commonly affected by the examined deadenylases in all three lines. This is in line with previous suggestions on the tissue-specific regulation of genes, at least in the case of CNOT6L [13,53], although not all deadenylases were studied and other explanations cannot be excluded.

We investigated whether one deadenylase affects the levels of other deadenylases. We silenced each of the studied deadenylases in turn, and measured the mRNA levels of the remaining enzymes. The results suggest that in NCI-H520 cells, deadenylases compensate for a silenced enzyme, as observed by the changes in their expression; as previously mentioned, two deadenylases, one each from the DEDD and EEP families, are present in the CCR4-NOT complex; CNOT7 or CNOT8 (DEDD), and CNOT6 or CNOT6L (EEP), respectively. CNOT8 is expressed in HEp-2 cells, but we are not able to detect it in NCI-H520 cells, possibly reflecting very low levels of expression. Moreover, this observation suggests that CNOT7 is the main DEDD deadenylase present in the CCR4-NOT complex in this cell type, possibly as a result of different expression regulation and compensation for the non-expressed CNOT8. According to the genetic network, CNOT6 and CNOT7 are the most prevalent and most interconnected nodes. Indeed, CNOT7 KD affects CNOT6 expression in NCI-H520 cells. PARN KD affects CNOT6L expression in both cell lines, while this was not observed in silico. Whether this implies a consequence of the dysregulated gene expression in cancer needs to be investigated further. 

It was shown that PARN targets a discrete set of mRNAs in mouse fibroblasts, many of which encode for the factors required in cell migration and adhesion, while analogous observations are reported in cell lines of cancer origin [27,55]. Furthermore, PARN is involved in the maturation of many non-coding RNAs, including small nucleolar RNAs (snoRNAs), small Cajal body-specific RNAs (scaRNAs), the telomerase RNA component, Y RNAs, microRNA-451, and microRNA-122 [56,57,58,59,60,61,62]. Nevertheless, in another report it is suggested that PARN and TOE1 (a deadenylase located mainly in the Cajal bodies) mediate the maturation of non-coding RNAs (ncRNAs) instead of modulating the length of mRNA poly(A) tails, and they act redundantly on scaRNAs in HeLa cells [54]. These reports suggest that PARN destabilizes both specific mRNA and miRNAs subsets. In line with the previous studies, our results show that PARN is associated with alterations in the levels of mRNAs (Figure 2), as well as with lncRNAs (Figure 4).

The members of the CNOT family were also investigated for their roles in gene expression. CNOT6 (Ccr4a) and CNOT6L (Ccr4b) have distinct roles in cell survival, the formation of P-bodies, and regulating gene expression in MCF7 cells. The knockdown of CNOT6 and/or CNOT6L reduced cell proliferation and cell survival, while gene expression profiling reveals that the enzymes regulate distinct gene sets associated with breast cancer cell proliferation, apoptosis, and the inhibition of tumor development [53]. The depletion of CNOT6L in cancer MCF7 cells and mouse NIH3T3 cells impairs cell proliferation, but highlights differences in the expression of specific genes assuming cell type-specific roles for the CNOT6 and CNOT6L [13,53]. Further studies from the Winkler lab, on the impact of CNOT7 and/or CNOT8 on gene expression profiles in KD MCF7 cells, highlight the partial redundancy between these two subunits of the CCR4-NOT complex, and the regulation of several genes contributing to cell proliferation [52].

The Table S8 summarizes the top up- and downregulated transcripts upon silencing. Of these transcripts, several commonly regulated samples are further discussed. In NCI-H520 cells, CIDEA (cell death-inducing factor DFFA-like effector A) is among the commonly upregulated transcripts that appears to be affected, with a high FC score for all silenced deadenylases of the CNOT family (Table S8). CIDEA is involved in apoptosis, while mice lacking functional CIDEA are resistant to obesity and diabetes; *CIDEA* mRNA is expressed in the white human fat cells and in brown mouse adipocytes, and low adipose CIDEA expression is associated with metabolic syndrome. Interestingly, the silencing of CIDEA stimulated lipolysis and increased TNF-alpha secretion, revealing a role of the factor in the regulation of lipolysis regulation and metabolic complications of obesity in humans, possibly mediated by cross-talk between CIDEA and TNF-alpha [63,64]. Among the factors studied in this work, NOC has an established role in obesity; mice lacking NOC are resistant to diet-reduced obesity, accompanied by deficits in lipid metabolism, as well as by changes in glucose and insulin sensitivity [65]. Interestingly, CIDEA is not among the top affected factors upon NOC silencing (Table S8). Taken together with the fact that NOC is considered a phosphatase, and has limited impact on the expression of deadenylases (Figure 5), it should be interesting to investigate for any overlapping activity of other deadenylase that destabilizes *CIDEA* mRNA. On the other hand, in NCI-H520 cells, *SULT1C4* (sulfotransferase 1C4) transcript is among the downregulated factors, with the highest FC score. SULTs catalyze the conjugation of a sulfonate moiety on a substrate, including hormones, neurotransmitters, sterols, and xenobiotics. They are classified into six gene families, and of the four, only two are present in humans; *SULT1* and *SULT2* family genes are expressed in the liver [66]. SULT1C4 belongs to the SULT1 family utilizing 3′-phospho-5′-adenylyl sulfate (PAPS) as a donor to sulfate conjugation of phenolic compounds. *SULT1C4* mRNA is abundant in prenatal human liver specimens, but SULT1C4 protein is barely detectable [67]. SULT1C4 seems to occur in two transcript variants in human liver, TV1 (full-length) and TV2 (lacking exons 3 and 4), and the discordance between mRNA and protein levels is attributed to the inability of the more abundant TV2 to produce stable protein [68]. In our analysis, we find that the SULT1C4 transcript is downregulated by all silenced deadenylases and NOC, suggesting common regulatory elements and mechanisms that determine its stability.

Of the deregulated transcripts with the highest FC scores in HEp-2 cells, MALAT1 from the up-, as well as HS6ST3, TM4SF20, and PLCL1 from the downregulated transcripts are discussed.

*MALAT1* (metastasis-associated lung adenocarcinoma transcript 1), also known as nuclear paraspeckle assembly transcript 2 *NEAT2* [69], is a long non-coding RNA. A cleavage and polyadenylation signal at the *MALAT1* locus generates a ~7.4 kb polyadenylated product in human, yet the latter represents less than 1% of the total transcripts in the cell [70]. That is because the mature 3′ end of *MALAT1* is mostly generated several hundred nucleotides upstream of the poly(A) site. Despite the distinct generation of its 3′ end, which differentiates it from the canonical polyadenylation processing, the mature *MALAT1* transcript does have a short 3′ end adenylate tract (<20 nt) [70]. *MALAT1* is dysregulated in many cancers, including non-small cell lung cancer [71,72] and modulates cell proliferation, apoptosis, migration, and invasion. In particular, in NSCLC, *MALAT1* is involved in STAT3, SRSF7, and PBOV1 (prostate and breast cancer overexpressed 1) pathways; *MALAT1* affects the levels of miR-124, miR-374b-5p, and miR-28-5p, which in turn, target *STAT3*, *SRSF7*, and *PBOV1* mRNAs [72,73,74], respectively. In our results, the *MALAT1* transcript shows an increased FC score in all three CNOT deadenylases and NOC in HEp-2 cells. How each deadenylase singularly, or in concert, modulates the levels of *MALAT1* mRNA, and any potential impact on pathways such as the above-mentioned STAT3, SRSF, and PBOV1 pathways, remain to be investigated.

HS6ST3 belongs to the above-mentioned SULT enzymes, in particular in the SULT6 family, which catalyzes the transfer of a sulfate group from PAPS to N-sulfoglucosamine (position 6) of heparan sulfate (HS). Sulfation has a major effect on the chemical and functional homeostasis of substrate chemicals. SULTs are widely expressed in metabolically active or hormonally responsive tissue, while alterations of SULTs are frequently observed in cancer [75]. HS6ST3 is strongly downregulated upon the silencing of all five factors in HEp-2 cells. As mentioned above, SULT1C4, another member of the SULT family, is strongly downregulated in NCI-H520 cells by all silenced deadenylases (Table S8), suggesting that members of the SULTs are common targets of deadenylases. Further research on regulatory factors, such as *cis*-elements on *SULT* transcripts and/or miRNAs, may reveal whether specific deadenylases destabilize selected *SULT* mRNAs, or if they have overlapping functions [1].

Our analyses in NCI-H520 and HEp-2 cells reveal several GOs enriched upon CNOT6, CNOT6L, CNOT7, and CNOT8 silencing, and a very limited number of transcripts commonly affected between two cell lines. Moreover, the observation that the examined deadenylases share no common targets between NCI-H50, HEp-2, and MCF7 cells imply that these enzymes target specific pathways, likely through differential regulation/expression and recruitment by specific factors in a tissue-dependent manner. Although CNOT6 and CNOT6L, and CNOT7 and CNOT8 have overlapping roles, due to a high similarity in their amino acid sequences [52], they play distinct roles [76,77,78]. Moreover, CNOT6/6L and CNOT7/8 have distinct biochemical functions in the complex [79,80].

## 4. Materials and Methods

### 4.1. Genetic Network

The genetic network for Caf1, Ccr4, CNOT6, CNOT6L CNOT7, CNOT8, and PARN was drawn using the GeneMANIA server, and by selecting all possible network interactions available. GeneMANIA is an online genetic network design and drawing tool, which enables the construction of gene interaction networks based on the relationships between genes, co-expression, physical interaction, and their potential functions as defined in the GeneMANIA server. The GeneMANIA label propagation algorithm uses a fast heuristic algorithm, the Gaussian field label propagation algorithm, which is derived from ridge regression, and differs in weighting compared to previous algorithms. This algorithm optimizes the network weights and calculates the discriminant values separately, thus, having the advantage of performing the computationally intensive label propagation only once. This algorithm’s difference lies in cases of prediction problems of the gene function, where the number of positive labeled genes is almost always only a very small percentage of the total number of genes. The GeneMANIA label propagation algorithm defines the unlabeled nodes’ initial bias as the average bias of the labeled nodes: n^+^ − n^−^/n^+^ + n^−^, where n^+^ is the number of positive, and n^−^ the number of negative, examples. This way of defining these label biases provides an improvement in the accuracy of predictions in these cases. Generally, this algorithm combines multiple input data sources fast enough to be employed on a web server, for integrating multiple functional association networks, and predicting gene function, from a single process-specific network using label propagation [37,38].

### 4.2. Cell Culture and Transfection

NCI-H520 and HEp-2 cell lines (ATCC) were cultured in RPMI-1640 and MEM, respectively (Biosera, Boussens, France), supplemented with 10% fetal bovine serum (FBS, Biosera) and 1% antibiotics (penicillin/streptomycin; Biosera), and were incubated at 37 °C in a 5% CO_2_ atmosphere. Cells were transfected with shRNAs against deadenylases control, using cationic liposomes (Xfect; Clontech, Mountain View, CA, USA), as previously described. All shRNAs were from Sigma-Aldrich (MISSION^®^ shRNA; Merck KGaA, Darmstadt, Germany). The following shRNA plasmids were used to silence the respective deadenylases: PARN (NM_002582), CNOT6 (NM_15455), CNOT6L (NM_012118), CNOT7 (NM_013354), CNOT8 (NM_004779), NOCTURNIN (NM_012118), and non-targeting control (SHC016). Twelve hours later, cells were selected with puromycin (6 μg/mL). All cell lines used in the study tested negative for mycoplasma.

### 4.3. RNA Extraction, Reverse Transcription, and qRT-PCR

Total RNA was isolated using the TRI Reagent^®^ Protocol (Sigma-Aldrich). RNA quantity and quality were determined using spectrophotometry (BioPhotometer Plus, Eppendorf, Wien, Austria). One microgram of the total RNA was reverse-transcribed using the PrimeScript RT-PCR kit (Clontech, Mountain View, CA, USA), according to the manufacturer’s instructions. The reaction mixture was incubated for 50 min at 42 °C, and terminated for 15 min at 70 °C. Quantitative polymerase chain reaction (qPCR) was performed on cDNA using KAPA SYBR Fast Universal qPCR kit (KAPA Biosystems, Cape Town, South Africa), and carried out in the Mx3005PTM real-time PCR system (Stratagene; Agilent Technologies Inc., Santa Clara, CA, USA) using the following conditions: 1 cycle at 95 °C for 3 min, followed by 40 cycles at 95 °C for 3 s, 60 °C for 30 s, and 72 °C for 11 s, and a final dissociation cycle at 95 °C for 60 s, and a progressive rise from 55 °C to 95 °C. The primer sequences used for the deadenylases were the ones previously described [11,23,27].

### 4.4. DNA Microarrays

Comprehensive gene expression analysis was performed at NIMGenetics (Madrid, Spain). Samples of NCI-H520 and HEp-2 cells upon deadenylase silencing were hybridized to microarrays SurePrint G3 Human GE 60 K Microarray, and Whole Human Genome Agilent 4 × 44 K oligo Microarray (Agilent Technologies, Inc., Santa Clara, CA, USA). To exclude any possible transcripts affected by the transfection procedure itself, we used non-target shRNA (Sigma), pLKO.1 empty vector (Sigma), and wild-type cells as controls of expression. Raw microarray data files are available in the GEO database with accession numbers GSE67536 and GSE67598 [50,51].

### 4.5. Bioinformatics Analysis

Venn diagrams were generated using the online freely available software “Venn Diagrams” (http://bioinformatics.psb.ugent.be/webtools/Venn/ (accessed on 21 March 2021). Venn diagrams included deregulated transcripts in NCI-H520 and HEp-2 cells upon deadenylases silencing, and the deregulated transcripts in MCF7 cells upon deadenylases silencing (E-MEXP-2926 and E-MEXP-2218) [52,53]. Gene ontology (GO) analysis was conducted using the ConsensusPathDB-human (http://consensuspathdb.org/) (accessed on 21 March 2021). A heat map of non-coding RNA transcripts affected by deadenylases (FC ≥ 2.0) was generated using GraphPad Prism (v8, La Jolla, CA, USA).

### 4.6. Statistical Analysis

Statistical analysis was performed using GraphPad Prism (v8, La Jolla, CA, USA). Relative gene expression was normalized to non-targeting shRNA. One-sample *t* test was used and the values *p* ≤ 0.001 (***), *p* ≤ 0.01 (**), and *p* ≤ 0.05 (*) were considered significant. All experiments were performed at least three times.

## 5. Conclusions

Conclusively, we designed a genetic network to exploit the variety of data associated with deadenylases, which highlighted the prevalence of CNOT6 and CNOT7. The subsequent microarray analysis data upon the silencing of specific deadenylases in two cell lines, NCI-H520 and HEp-2, as well as comparison with published data from MCF7 cells, show that specific deadenylases target unique subsets of mRNAs, while the levels of several mRNAs are altered in common by multiple deadenylases, thus implying overlapping functions for the enzymes. The silencing of each deadenylase affects the expression of other deadenylases. Together with the previous observations, as well as the fact that CNOT8 is not detected in one of the examined cell lines, our study supports the suggestion that deadenylases have overlapping roles in a tissue-specific manner; the absence of a deadenylase is compensated by other enzymes to mediate destabilization of RNAs. Finally, several non-coding RNA genes are affected, either specifically or in common, by the deadenylases examined, thus expanding the involvement of deadenylases beyond mRNA turnover. The data presented here encourages the study of the role of deadenylases in other cancer types, such as small-cell lung cancer, and their potential as biomarkers on clinical practice towards new targeted therapies in the future.

## Data Availability

Data available from the corresponding author on reasonable request.

## Supplementary Materials

The following are available online at https://www.mdpi.com/article/10.3390/molecules27103102/s1, Table S1: Upregulated and downregulated transcripts upon silencing of CNOT6, CNOT6L, CNOT7, PARN, and NOC in NCI-H520 cells. Table S2: Over-representation analysis of common differentially overexpressed and downregulated transcripts after CNOT6, CNOT6L, and CNOT7 silencing in NCI-H520 cells, Table S3: Upregulated and downregulated transcripts (FC ≥ 2.0) upon silencing of CNOT6, CNOT7, CNOT8, PARN, and NOC in HEp-2cells, Table S4: Over-representation analysis of common differentially overexpressed and downregulated transcripts after CNOT6, CNOT6L, and CNOT7 silencing in HEp-2 cells, Table S5: Common transcripts upon silencing of CNOT6, CNOT6L, CNOT7, PARN, and NOC in NCI-H520, HEp-2, and MCF7 cells (FC ≥ 1.5), Table S6: Upregulated and downregulated non-coding RNAs upon silencing of CNOT6, CNOT6L, CNOT7, PARN, and NOC in NCI-H520 cells (FC ≥ 2.0), Table S7: Upregulated and downregulated non-coding RNAs upon silencing of CNOT6, CNOT7, CNOT8, PARN, and NOC in HEp-2 cells (FC ≥ 2.0), Table S8: Top ten upregulated and downregulated transcripts upon silencing of CNOT6, CNOT7, CNOT8, PARN, and NOC in NCI-H520 and HEp-2 cells.

## Sample Availability

No biological/clinical samples were used in the study.
