# Supplementary material for: Integrated Deadenylase Genetic Association Network and Transcriptome Analysis in Thoracic Carcinomas
Source: Molecules. 2022 May 12;27(10):3102. doi: 10.3390/molecules27103102 (PMC9145511; doi:10.3390/molecules27103102)
Supplement: Supplementary file 1 [file molecules-27-03102-s001.zip › Table S2.pdf]

Table S2. Over-Representation Analysis of common differentially overexpressed and downregulated transcripts after CNOT6, CNOT6L and CNOT7 silencing in NCI-H520 cells.

Gene Ontology Term	Category, Level	Set Size	Candidates Contained	p-Value	q-Value
**GOs from the 23 commonly upregulated in NCI-H520 cells (FC ≥ 2)**					
GO:0099634 postsynaptic specialization membrane	CC 2	101	3 (3.0%)	7.42e-05	0.00208
GO:0033038 bitter taste receptor activity	MF 5	25	2 (8.0%)	0.000191	0.000574
GO:0008527 taste receptor activity	MF 4	31	2 (6.5%)	0.000296	0.00236
GO:0097106 postsynaptic density organization	BP 5	32	2 (6.2%)	0.000315	0.00315
GO:0099084 postsynaptic specialization organization	BP 4	35	2 (5.7%)	0.000377	0.0083
GO:0004888 transmembrane signaling receptor activity	MF 3	1295	6 (0.5%)	0.000403	0.00403
GO:0050877 nervous system process	BP 3	1426	6 (0.4%)	0.000676	0.0223
GO:0050912 detection of chemical stimulus involved in sensory perception of taste	BP 5	47	2 (4.3%)	0.000682	0.00341
GO:0007600 sensory perception	BP 4	978	5 (0.5%)	0.000905	0.00995
GO:0038023 signaling receptor activity	MF 2	1513	6 (0.4%)	0.000927	0.00927
GO:0003008 system process	BP 2	2136	7 (0.3%)	0.000932	0.0214
GO:0016459 myosin complex	CC 2	66	2 (3.0%)	0.00134	0.0127
GO:0099060 integral component of postsynaptic specialization membrane	CC 3	74	2 (2.7%)	0.00168	0.014
GO:0098839 postsynaptic density membrane	CC 3	75	2 (2.7%)	0.00173	0.014
GO:0031594 neuromuscular junction	CC 2	76	2 (2.6%)	0.00177	0.0127
GO:0098948 intrinsic component of postsynaptic specialization membrane	CC 2	77	2 (2.6%)	0.00182	0.0127
GO:0045211 postsynaptic membrane	CC 3	323	3 (0.9%)	0.00221	0.014
GO:0099572 postsynaptic specialization	CC 2	343	3 (0.9%)	0.00263	0.0147
GO:0050808 synapse organization	BP 3	391	3 (0.8%)	0.0038	0.0605
GO:0099055 integral component of postsynaptic membrane	CC 4	117	2 (1.7%)	0.00414	0.029
GO:0098936 intrinsic component of postsynaptic membrane	CC 3	122	2 (1.6%)	0.00449	0.0213
GO:0097060 synaptic membrane	CC 2	430	3 (0.7%)	0.00496	0.0231
GO:0004930 G protein-coupled receptor activity	MF 4	880	4 (0.5%)	0.00504	0.0201
GO:0050907 detection of chemical stimulus involved in sensory perception	BP 4	484	3 (0.6%)	0.00689	0.0505
GO:0099699 integral component of synaptic membrane	CC 3	155	2 (1.3%)	0.00715	0.0272
GO:0099173 postsynapse organization	BP 3	162	2 (1.2%)	0.00778	0.0605
GO:0099240 intrinsic component of synaptic membrane	CC 2	167	2 (1.2%)	0.00825	0.033
GO:0009593 detection of chemical stimulus	BP 3	520	3 (0.6%)	0.00839	0.0605
GO:0007606 sensory perception of chemical stimulus	BP 5	534	3 (0.6%)	0.00902	0.0301
GO:0050906 detection of stimulus involved in sensory perception	BP 3	537	3 (0.6%)	0.00916	0.0605
**GOs from the 33 commonly downregulated in NCI-H520 cells (FC ≥ 2)**					
**Gene Ontology Term**	**Category, Level**	**Set Size**	**Candidates Contained**	**p-Value**	**q-Value**
GO:0099529 neurotransmitter receptor activity involved in regulation of postsynaptic membrane potential	MF 5	51	2 (3.9%)	0.000908	0.00469
GO:0098960 postsynaptic neurotransmitter receptor activity	MF 4	53	2 (3.8%)	0.00098	0.00536
GO:0022835 transmitter-gated channel activity	MF 4	62	2 (3.2%)	0.00134	0.00536
GO:0022824 transmitter-gated ion channel activity	MF 5	62	2 (3.2%)	0.00134	0.00469

GO:0099060	integral component of postsynaptic specialization membrane	CC 3	**74**	**2 (2.7%)**	0.0019	0.0439
GO:0098948	intrinsic component of postsynaptic specialization membrane	CC 2	**77**	**2 (2.6%)**	0.00206	0.0491
GO:0098916	anterograde trans-synaptic signaling	BP 5	**697**	**4 (0.6%)**	0.00276	0.0469
GO:0099537	trans-synaptic signaling	BP 4	**705**	**4 (0.6%)**	0.00287	0.0569
GO:0007215	glutamate receptor signaling pathway	BP 4	**92**	**2 (2.2%)**	0.00292	0.0569
GO:0099536	synaptic signaling	BP 3	**711**	**4 (0.6%)**	0.00296	0.119
GO:0099634	postsynaptic specialization membrane	CC 2	**101**	**2 (2.0%)**	0.0035	0.0491
GO:0030659	cytoplasmic vesicle membrane	CC 4	**767**	**4 (0.5%)**	0.0039	0.035
GO:0012506	vesicle membrane	CC 3	**788**	**4 (0.5%)**	0.00429	0.0439
GO:0030594	neurotransmitter receptor activity	MF 3	**117**	**2 (1.7%)**	0.00467	0.0327
GO:0099055	integral component of postsynaptic membrane	CC 4	**117**	**2 (1.7%)**	0.00467	0.035
GO:0098936	intrinsic component of postsynaptic membrane	CC 3	**122**	**2 (1.6%)**	0.00507	0.0439
GO:0015276	ligand-gated ion channel activity	MF 5	**139**	**2 (1.4%)**	0.00653	0.0152
GO:0060078	regulation of postsynaptic membrane potential	BP 4	**142**	**2 (1.4%)**	0.0068	0.0815
GO:0099699	integral component of synaptic membrane	CC 3	**155**	**2 (1.3%)**	0.00806	0.0524
GO:0055085	transmembrane transport	BP 4	**1520**	**5 (0.3%)**	0.00836	0.0815
GO:0099240	intrinsic component of synaptic membrane	CC 2	**167**	**2 (1.2%)**	0.0093	0.0868

CC cellular component. BC biological process. MF molecular function.
