# Supplementary material for: Integrated Deadenylase Genetic Association Network and Transcriptome Analysis in Thoracic Carcinomas
Source: Molecules. 2022 May 12;27(10):3102. doi: 10.3390/molecules27103102 (PMC9145511; doi:10.3390/molecules27103102)
Supplement: Supplementary file 1 [file molecules-27-03102-s001.zip › Table S4.pdf]

Table S4. Over-Representation Analysis of common differentially overexpressed and downregulated transcripts after CNOT6, CNOT6L and CNOT7 silencing in HEp-2 cells.

**GOs from the 6 commonly upregulated in HEp-2 cells (FC ≥ 2)**					
**Gene ontology term**	**Category, Level**	**Set Size**	**Candidates Contained**	**p-Value**	**q-Value**
GO:0016811 hydrolase activity, acting on carbon-nitrogen (but not peptide) bonds, in linear amides	MF 4	**92**	**2 (2.2%)**	6.72e-05	6.72e-05
GO:0016810 hydrolase activity, acting on carbon-nitrogen (but not peptide) bonds	MF 3	**150**	**2 (1.3%)**	0.000179	0.000179
GO:0060548 negative regulation of cell death	BP 5	**977**	**2 (0.2%)**	0.00742	0.0148
**GOs from commonly downregulated in HEp-2 cells (FC ≥ 2)**					
**Gene Ontology Term**	**Category, Level**	**Set Size**	**Candidates Contained**	**p-Value**	**q-Value**
GO:0034483 heparan sulfate sulfotransferase activity	MF 5	**15**	**2 (13.3%)**	0.000941	0.0103
GO:0016881 acid-amino acid ligase activity	MF 4	**20**	**2 (10.0%)**	0.00169	0.0371
GO:0006029 proteoglycan metabolic process	BP 5	**88**	**3 (3.4%)**	0.00248	0.233
GO:0001578 microtubule bundle formation	BP 4	**89**	**3 (3.4%)**	0.00256	0.258
GO:0006935 chemotaxis	BP 3	**635**	**7 (1.1%)**	0.00313	0.365
GO:0042330 taxis	BP 2	**638**	**7 (1.1%)**	0.00321	0.0565
GO:0048870 cell motility	BP 2	**1623**	**12 (0.7%)**	0.00327	0.0565
GO:0051674 localization of cell	BP 2	**1623**	**12 (0.7%)**	0.00327	0.0565
GO:0006928 movement of cell or subcellular component	BP 2	**2079**	**14 (0.7%)**	0.00338	0.0565
GO:0050920 regulation of chemotaxis	BP 4	**208**	**4 (1.9%)**	0.00376	0.258
GO:0051272 positive regulation of cellular component movement	BP 5	**514**	**6 (1.2%)**	0.00474	0.233
GO:0040017 positive regulation of locomotion	BP 4	**533**	**6 (1.1%)**	0.00564	0.258
GO:0062023 collagen-containing extracellular matrix	CC 3	**374**	**5 (1.3%)**	0.00566	0.203
GO:0099513 polymeric cytoskeletal fiber	CC 4	**715**	**7 (1.0%)**	0.00596	0.179
GO:0009100 glycoprotein metabolic process	BP 4	**403**	**5 (1.2%)**	0.00763	0.258
GO:0051828 entry into other organism involved in symbiotic interaction	BP 4	**135**	**3 (2.2%)**	0.00818	0.258
GO:0044409 entry into host	BP 5	**135**	**3 (2.2%)**	0.00818	0.233
GO:0051806 entry into cell of other organism involved in symbiotic interaction	BP 5	**135**	**3 (2.2%)**	0.00818	0.233
GO:0005874 microtubule	CC 5	**411**	**5 (1.2%)**	0.00835	0.0751
GO:0001935 endothelial cell proliferation	BP 3	**143**	**3 (2.1%)**	0.00957	0.365

CC cellular component. BC biological process. MF molecular function.
